# Erythema Ab Igne: A Rare Presentation of Toasted Skin Syndrome With the Use of a Space Heater

**DOI:** 10.7759/cureus.13401

**Published:** 2021-02-17

**Authors:** Zarah Haleem, Judith Philip, Safwan Muhammad

**Affiliations:** 1 College of Medicine, American University of Antigua, Coolidge, ATG; 2 Internal Medicine, University of Maryland Medical Center Midtown Campus, Baltimore, USA

**Keywords:** squamous cell carcinoma, erythema ab igne, space heater, toasted skin syndrome, merkel cell carcinoma, cutaneous hyperpigmentation, atypical rash

## Abstract

Erythema ab igne, also known as toasted skin syndrome, is an acquired asymmetric hyperpigmented dermatosis that is caused by repeated exposure to moderate heat or infrared radiation. Hyperpigmentation is caused by the degeneration of elastic fibers and basal cells resulting in the release of melanin. Historically found in bakers and industrial workers, this condition has recently resurfaced in medical literature with the use of novel heat sources such as laptops and heated car seats. While this condition can resolve spontaneously after removal of heat exposure, delay in diagnosis and persistent exposure can lead to permanent pigmentation or progression to Merkel cell carcinoma, basal cell carcinoma, and squamous cell carcinoma.

## Introduction

Toasted skin syndrome is a rare hyperpigmented dermatosis that can occur at any site with recurrent exposure to heat or an infrared source. This condition is known to be more prevalent in women than men and patients with chronic pain [1-3]. The pathophysiology does not seem to be fully understood and multiple mechanisms have been proposed, including the repeated heat exposure damaging superficial blood vessels leading to hemosiderin deposition and subsequent hyperpigmentation. Additionally, the release of melanin from heat-induced damage to elastic fibers and basal cells imparts the characteristic reticular rash in a vascular pattern [4]. While it usually resolves in weeks to months after the removal of the heat source, the rash has a propensity to become permanent, and in some cases, transforms into cutaneous malignancies [5-7]. Both patients and providers must be aware of the association of this rash with repeated heat exposure. We present a unique case of erythema ab igne from a novel heat source, a space heater.

This case was previously presented as a poster at the ACP Internal Medicine Meeting on April 23, 2020.

## Case presentation

A 54-year-old female with a past medical history of peripheral vascular disease presented with a skin rash on the abdomen and right arm that started six months ago. She had a similar rash on her left arm which had spontaneously resolved over the course of a few weeks. The rash was not amenable to over-the-counter triamcinolone 0.1% ointment. The patient was a retired contractor who worked indoors with some exposure to steel, iron, aluminum, and brass. She denied pruritus or burning over the rash, allergies, a family or childhood history of any rashes, exposure to rodents, exacerbation with sunlight, recent travel, occupational exposures, outdoor activities, or recent medication changes. She reported marijuana use amounting to 1 g daily over the past year. On further questioning, the patient stated that she was “constantly cold” with intermittent episodes of hot flashes. She reported using a space heater under her blanket, directly over her skin on the right side of her abdomen continuously throughout the day for comfort, and frequently took hot water baths each lasting approximately 30 minutes.

**Figure 1:**
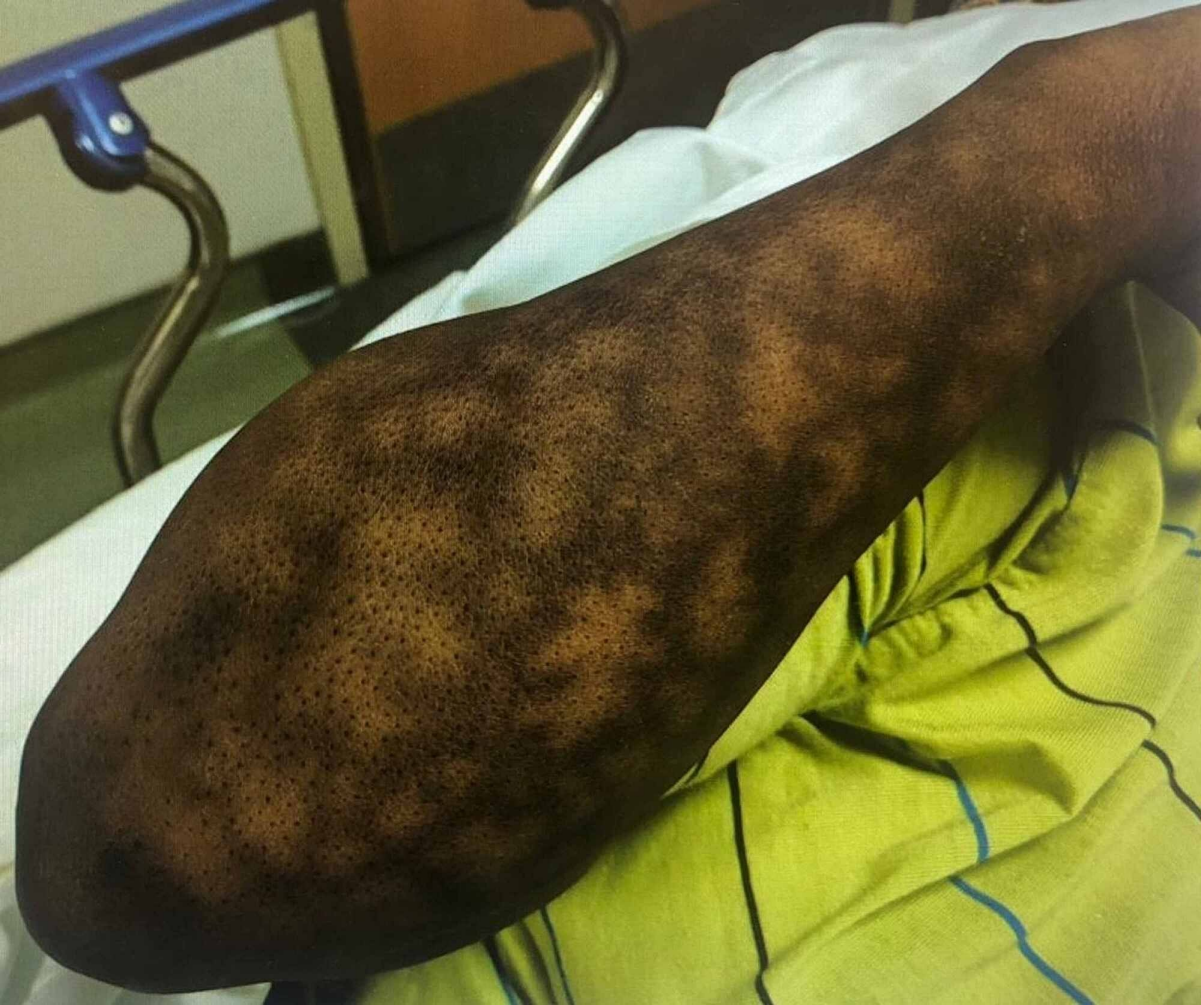
Right forearm with hyperpigmented, erythematous, non-blanching, reticular rash.

**Figure 2:**
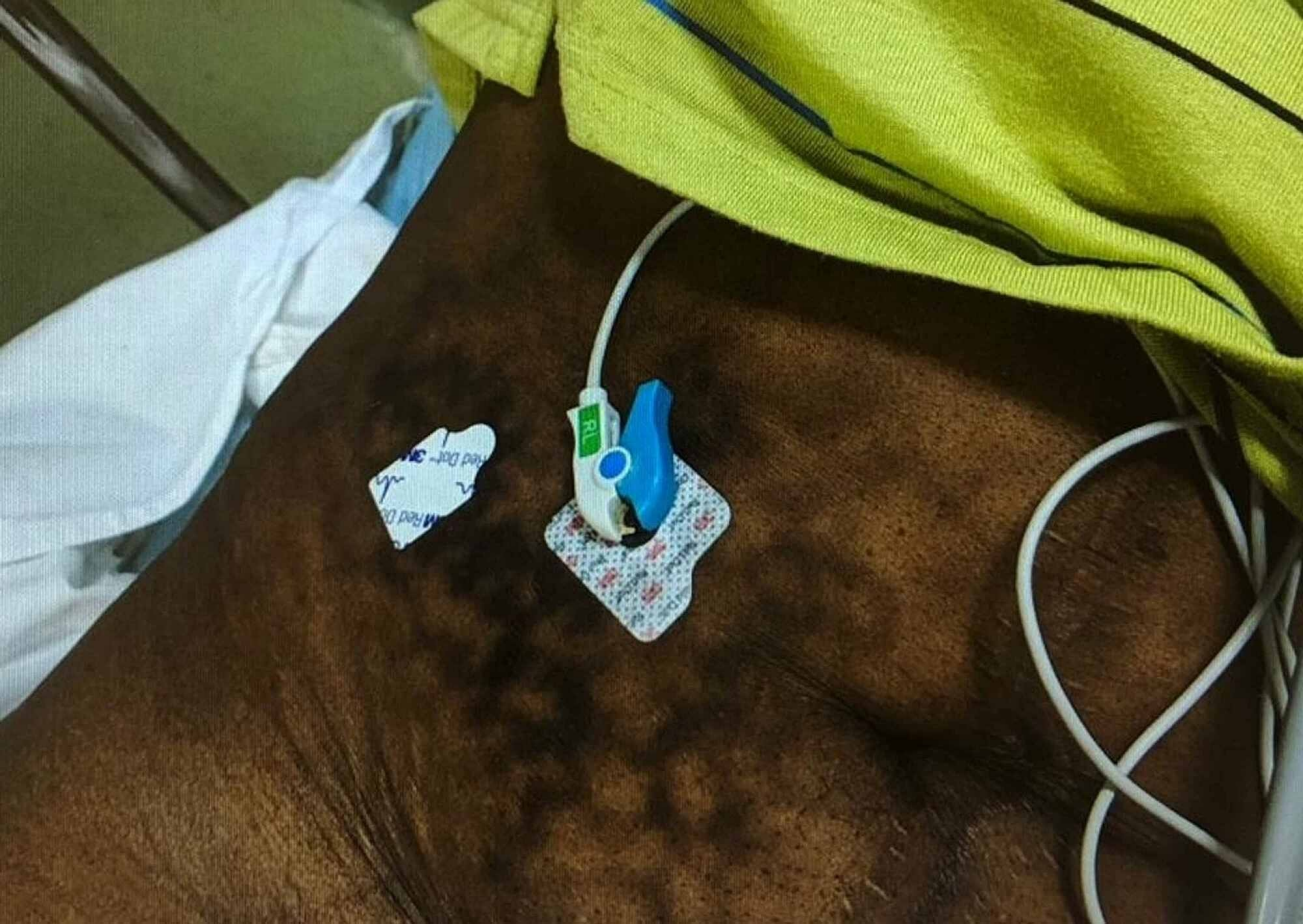
Right lower quadrant of the abdomen with erythema ab igne.

Examination of the skin revealed a hyperpigmented, erythematous, non-blanching, and reticular rash on her right forearm and right lower quadrant of the abdomen (Figures 1, 2). Her vitals were within normal limits. Laboratory workup revealed normal blood count, electrolytes, and coagulation profile. Workup for hypothyroidism showed thyroid-stimulating hormone 0.16 mIU/L (0.47-4.68 mIU/L), free T4 ng/dL 1.49 (0.8-1.8 ng/dL), and a negative human immunodeficiency virus status. Further testing revealed erythrocyte sedimentation rate of 20 mm/hr (4-30 mm/hr), anti-Scl-70 autoantibodies 1.2 U (<20 U), anti-SS-A IgG autoantibodies 2.3 U (<20 U), anti-SS-B IgG autoantibodies 3.3 U (<20 U) and negative antinuclear antibodies, urinary metanephrines, and 5-hydroxyindoleacetic acid. Computerized tomography scans of the abdomen and chest did not reveal an abnormal mass or fluid accumulation. The patient was clinically diagnosed with erythema ab igne given her extensive history of heat exposure to the affected areas and characteristic appearance of the rash in the setting of a negative workup. On follow-up two months after discharge, she noted the discoloration had improved with the reduction of heat exposure.

## Discussion

Erythema ab igne is a reticular, hyperpigmented rash that is acquired from moderate heat and infrared exposure, with temperature often ranging from 43 to 47°C [2]. In literature, it has been given many terms, including “toasted skin syndrome” and “fire stains.” Historically, this condition was found to be more common among people with close, long-term exposure to heat sources such as fireplaces and bakeries. Chronic infrared heat exposure results in the degeneration of the elastic fibers and basal cells imparting the dark lacy discoloration with the release of melanin. The histopathological changes include epidermal atrophy, hyperkeratosis and parakeratosis, liquefactive degeneration of the basal layer, melanin and hemosiderin deposition, and the formation of telangiectasias. The dermis shows melanophages and isolated elastic fiber alterations similar to actinic elastosis, which is usually seen with prolonged sun exposure [4]. A rare variant includes crusting bullous lesions overlying the reticular rash. Though it is a benign rash that resolves quickly within a few weeks to months, rare complications decades after exposure include transformation into cutaneous squamous cell carcinoma, Merkel cell carcinoma, and heat-induced basal cell carcinoma in regions of the body without direct sun exposure, such as the perineum [5-7]. Common differential diagnoses include livedo reticularis, autoimmune connective tissue disorders, cutis marmorata, and livedo racemosa [8].

Although it has become less common since the availability of the central heating system, some continue to experience it from traditional sources. Interestingly, this rash has found its way back into literature and started to gain attention when it was described as laptop-induced erythema ab igne noted on a patient’s thighs following prolonged exposure to heat emanating from the laptop [9,10]. Similarly, it is noticed in those using sauna belts for abdominal obesity and those using a heat source for the management of pain [11]. Most of these heating devices are available over the counter such as sauna belts, heated blankets, and heated car seats. Skin lesions may not appear immediately after exposure, taking up to a few weeks or months. The diagnosis is made clinically with a history of heat exposure, but if equivocal, the rash will stain positive with Verhoeff-van Gieson elastic stain [12].

Biopsy is recommended if skin findings such as telangiectasias, nodules, or ulcers are suspicious for cancer. The mainstay of treatment is to identify the source of heat radiation and to avoid further exposure. Patient education and reassurance should be provided. The rash should be periodically monitored for early detection of alarming signs. In addition, topical bioflavonoids, topical 5-fluorouracil reducing keratinocyte dysplasia, and oral mesoglycan with their antithrombotic and profibrinolytic action have been effective for further management. Refractory cases have been treated with photothermolysis using a neodymium-doped yttrium aluminum garnet, ruby, or alexandrite laser therapy [2,13].

## Conclusions

This case of erythema ab igne emphasizes the significance of early identification with a thorough history and physical examination as treatment is simple and the rash is generally reversible. Physicians and patients should be aware of both typical and atypical sources of heat and infrared radiation including commercially available space heaters, heated blankets, and vests, which can cause erythema ab igne. Although rare, the prolonged and unchecked exposure from these sources can result in permanence or malignant transformation.
